# The implications of three major new trials for the effect of water, sanitation and hygiene on childhood diarrhea and stunting: a consensus statement

**DOI:** 10.1186/s12916-019-1410-x

**Published:** 2019-08-28

**Authors:** Oliver Cumming, Benjamin F. Arnold, Radu Ban, Thomas Clasen, Joanna Esteves Mills, Matthew C. Freeman, Bruce Gordon, Raymond Guiteras, Guy Howard, Paul R. Hunter, Richard B. Johnston, Amy J. Pickering, Andrew J. Prendergast, Annette Prüss-Ustün, Jan Willem Rosenboom, Dean Spears, Shelly Sundberg, Jennyfer Wolf, Clair Null, Stephen P. Luby, Jean H. Humphrey, John M. Colford

**Affiliations:** 1 Department of Disease Control, Faculty of Infectious Tropical Disease, London School of Hygiene and Tropical Medicine, London, UK; 2 Division of Epidemiology and Biostatistics, School of Public Health, University of California, Berkeley, CA USA; 3 Bill and Melinda Gates Foundation, Seattle, WA USA; 4 Department of Environmental Health, Rollins School of Public Health, Emory University, NE Atlanta, GA USA; 5 Department of Public Health, World Health Organization, Geneva, Switzerland; 6 Department of Agricultural and Resource Economics, North Carolina State University, Raleigh, NC USA; 7 Department of Civil Engineering, University of Bristol, Queens Building, Bristol, UK; 8 The Norwich Medical School, University of East Anglia, Norwich, UK; 9 Department of Civil and Environmental Engineering, Tufts University, Medford, MA USA; 10 Blizard Institute, Queen Mary University of London, London, UK; 11 Department of Economics, The University of Texas at Austin, Austin, TX USA; 12 Center for International Policy Research and Evaluation, Mathematica Policy Research, Washington, DC USA; 13 Department of Infectious Diseases and Geographic Medicine, Stanford University, Stanford, CA USA; 14 Department of International Health, Bloomberg School of Public Health, Johns Hopkins University, Baltimore, MD USA

**Keywords:** Diarrhea, Undernutrition, Stunting, Water, Sanitation, Hygiene

## Abstract

**Background:**

Three large new trials of unprecedented scale and cost, which included novel factorial designs, have found no effect of basic water, sanitation and hygiene (WASH) interventions on childhood stunting, and only mixed effects on childhood diarrhea. Arriving at the inception of the United Nations’ Sustainable Development Goals, and the bold new target of safely managed water, sanitation and hygiene for all by 2030, these results warrant the attention of researchers, policy-makers and practitioners.

**Main body:**

Here we report the conclusions of an expert meeting convened by the World Health Organization and the Bill and Melinda Gates Foundation to discuss these findings, and present five key consensus messages as a basis for wider discussion and debate in the WASH and nutrition sectors. We judge these trials to have high internal validity, constituting good evidence that these specific interventions had no effect on childhood linear growth, and mixed effects on childhood diarrhea. These results suggest that, in settings such as these, more comprehensive or ambitious WASH interventions may be needed to achieve a major impact on child health.

**Conclusion:**

These results are important because such basic interventions are often deployed in low-income rural settings with the expectation of improving child health, although this is rarely the sole justification. Our view is that these three new trials do not show that WASH in general cannot influence child linear growth, but they do demonstrate that these specific interventions had no influence in settings where stunting remains an important public health challenge. We support a call for transformative WASH, in so much as it encapsulates the guiding principle that – in any context – a comprehensive package of WASH interventions is needed that is tailored to address the local exposure landscape and enteric disease burden.

**Electronic supplementary material:**

The online version of this article (10.1186/s12916-019-1410-x) contains supplementary material, which is available to authorized users.

## Background

Recently, the results of three large factorial randomized controlled trials (RCTs) of water, sanitation and hygiene (WASH) interventions were published [1–3]. These three studies – referred to as the WASH-Benefits Bangladesh (WASH-B Bangladesh), the WASH-Benefits Kenya (WASH-B Kenya) and the Sanitation Hygiene Infant Nutrition Efficacy (SHINE) trials – were each conducted in a low-income rural setting with a high burden of stunting, and the WASH interventions delivered were very similar. All three evaluated the effects of these interventions on childhood diarrhea and linear growth, both independently and when combined with standard nutrition interventions. All three studies found no effect of any WASH intervention on child linear growth, and only mixed effects on diarrhea across the sites.

The studies were all cluster-based randomized controlled trials employing a factorial design to permit the evaluation of both the independent and combined effects of WASH and nutrition interventions on the outcomes of interest. Consenting pregnant women residing in the study areas were enrolled, together with their children in utero, and then followed up for between 18 and 24 months. A variety of health outcomes were assessed, including diarrhea prevalence and child growth (length-for-age z-scores). While the ‘treatment’ was allocated at a cluster level, typically forming one or two villages, the WASH interventions were delivered at the level of the household or immediate compound (typically two or three households) within which the enrolled children were born. As such, little change in community level coverage was effected as the index households or immediate compounds accounted for only a small fraction of the total number of households within a given cluster or community.

These studies adhered to best practice guidelines for human participant research [4], with pre-registration of trials (clinicaltrials.gov: NCT01590095, NCT01704105, NCT01824940), published protocols, and pre-specified analysis plans [5, 6]. The protocols included detailed measurement strategies with objective health outcomes and were adequately powered to detect small differences between arms. Active control arms were employed in two [2, 3] of the three trials, and data were managed remotely and analysed in duplicate by blinded statisticians.

The low-cost WASH interventions evaluated are typical of those often featuring in policy and programs in rural settings in low-income countries (LICs). All three included interventions to increase chlorination of drinking water at the point-of-use, to increase access to, and use of, ‘improved’ pit latrines, including the safe disposal of child feces; and to increase handwashing with soap by providing ‘handwashing stations’ with an ongoing supply of soap (Table 1). The implementation fidelity was high, with all interventions delivered as per protocol, and high compliance facilitated by regular provision of free commodities and supported by contextually appropriate, theory-based behavior change communication delivered to participants during regular home visits.

**Table 1:** Summary description of water, sanitation and hygiene (WASH) and nutrition interventions evaluated under the WASH-Benefits and SHINE trials

Trial	Water	Sanitation	Hygiene	Nutrition
WASH-Benefits trial, Bangladesh [7]				
Intervention arm	Water chlorination and promotion	Latrine improvements and promotion	Handwashing stations with soap and hygiene promotion	Nutrient supplementation and promotion
WASH SDG classification [7]	n/a	Basic	Basic	n/a
Details of intervention	A 10-L storage vessel with supply of disinfectant tablets	An ‘improved’ two-pit water-sealed latrine, plus potties and child stool collection device	Two handwashing stations per household, near latrine and kitchen, with regular supply of soap	Daily small-quantity of lipid-based nutrient supplement and promotion of appropriate and safe complementary feeding
WASH-Benefits trial, Kenya [8]				
Intervention arm	Water chlorination and promotion	Latrine improvements and promotion	Handwashing station with soap, and hygiene promotion	Nutrient supplementation and promotion
WASH SDG classification [7]	n/a	Basic	Basic	n/a
Details of intervention	Communal chlorine dispenser and supply of bottled chlorine	An ‘improved’ single pit latrine with plastic slab and hole-lid, plus potty and child stool collection device	Two handwashing stations per household, near latrine and kitchen, and quarterly supply of soap	Daily small-quantity of lipid-based nutrient supplement and promotion of appropriate and safe complementary feeding
SHINE trial, Zimbabwe [9]				
Intervention arm	Water chlorination and promotion	Latrine construction and promotion	Hand-washing stations with soap and hygiene promotion	Nutrient supplementation and promotion
WASH SDG classification [7]	n/a	Basic	Basic	n/a
Details of intervention	Monthly delivery of chlorine solution	A ventilated improved pit latrine constructed	Two handwashing stations per household, near latrine and kitchen, and monthly delivery of soap	Daily small-quantity of lipid-based nutrient supplement and promotion of appropriate and safe complementary feeding

Abbreviations: *n/a* not applicable, *SDG* Sustainable Development Goal, *WASH* water, sanitation and hygiene

The results of these trials arrive at the inception of an ambitious new WASH Sustainable Development Goal (SDG) that calls for, “universal access to safe and affordable drinking water and adequate and equitable sanitation and hygiene for all by 2030” [7]. These results also come at a time when calls are being made for the further integration of WASH across multiple health sectors, including nutrition [8] but also others such as neglected tropical diseases [9], and maternal and neonatal health [10]. Against this backdrop, and in response to these findings, the World Health Organization (WHO) and the Bill and Melinda Gates Foundation (BMGF) convened an expert meeting of researchers to consider the implications of this new evidence for WASH policy and research.

## Consensus messages

Here, we distil the salient points of consensus from the meeting into five key messages.

### 1. Despite high compliance, the evaluated WASH interventions – as delivered in these settings – had no effect on linear growth, and mixed effects on diarrhea

**Fig. 1:**
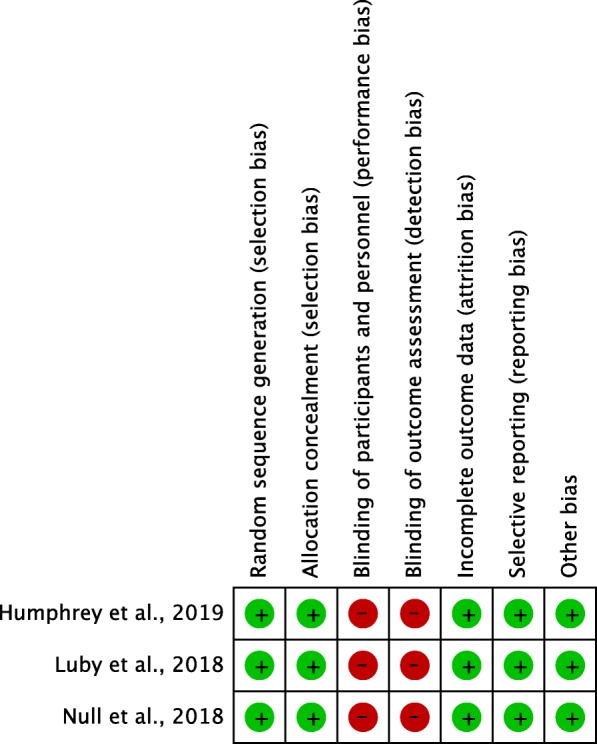
Cochrane risk of bias assessment for the WASH-Benefits and SHINE trials

We judge these trials to have high internal validity (Fig. 1; full table in Additional file 1), constituting good evidence that these specific interventions – as delivered in these settings – had no effect on childhood linear growth, and mixed effects on childhood diarrhea. Our view is that fidelity and compliance were at least as high as what might reasonably be expected in a typical WASH project or program.

**Fig. 2:**
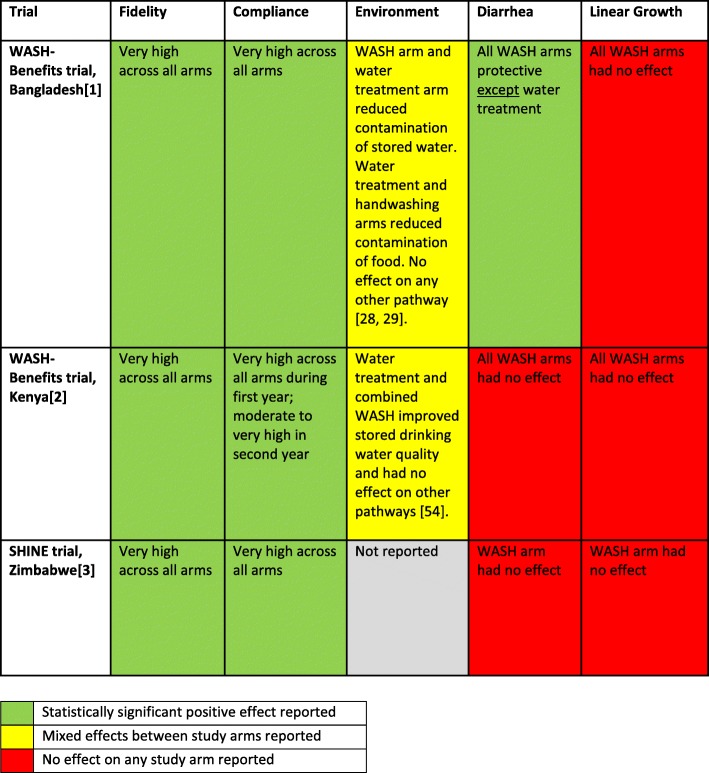
Summary of key reported results for the WASH-B and SHINE trials

In all three trials, these basic WASH interventions had no effect on linear growth (Fig. 2). The high validity of these studies and the consistent effects across three separate sites constitute good quality evidence that these basic WASH interventions, as delivered in these settings, did not reduce stunting. In addition, the novel factorial design of these trials provides good evidence that these WASH interventions in these populations offered no additonal benefit to the evaluated nutrient supplementation intervention as delivered alone. Whilst the effects on linear growth were consistent the underlying reasons for this lack of effect may differ between settings.

The observed effects on diarrhea were mixed, ranging from no effect in Kenya [2] and Zimbabwe [3], to a large relative risk reduction in Bangladesh – albeit against a much lower baseline prevalence [1]. These differences could be the result of interactions between the interventions and features of the study setting and/or population; for example, the local etiologies of diarrheal disease, pre-intervention WASH conditions, the relative importance of different environmental transmission pathways for diarrhea, and the relative importance of zoonotic agents of infection. Recent studies demonstrate the diversity of diarrheal disease etiology across settings and age groups [11, 12], with the transmission of different pathogens more or less likely to be interrupted by basic WASH interventions. For example, *Cryptosporidium*, a well-established waterborne cause of both endemic [11] and epidemic diarrhea [13], is highly chlorine resistant, thereby likely rendering chlorination, as evaluated in these trials, ineffective [14].

As pointed out by numerous researchers over the decades, different environmental settings require different WASH interventions [15], and the same interventions may even have different effects on health in the same settings at different times [16]. In grossly contaminated environments, where childhood exposure to a variety of enteric pathogens occurs through multiple environmental pathways, partial or even absolute elimination of a single pathway may yield no health benefit. At the same time, under different conditions, small incremental gains may, in some cases, prove catalytic [17, 18]. Alternatively, while some interventions may fail to significantly reduce endemic diarrheal disease, they may still offer protection against epidemic diarrheal disease events [1].

### 2. The biological plausibility of WASH as public health interventions is not challenged by these findings

It is well-established that contact with human feces is hazardous to human health: human feces contain various disease-inducing viruses, bacteria, protozoa, and other parasites [19]. Ingestion of these microorganisms in sufficient quantity has been demonstrated to cause disease in decades of challenge studies for a range of pathogens, e.g. *Vibrio cholera*e [20], *Shigella* [21], and *Campylobacter* [22]. Fecal–oral transmission of these pathogens can occur by multiple environmental pathways [23], and all WASH interventions can plausibly prevent some fraction of that transmission. This logic is not challenged by these findings, but the mixed results for diarrhea suggest that these interventions had heterogenous effects on childhood environmental exposure to enteric pathogens [24].

Two of the three trials [1, 2] published ancillary studies to assess the effects of the intervention on environmental contamination [25–27]. They did this by quantifying fecal indicator bacteria (*Escherichia coli*) in environmental media corresponding to environmental transmission pathways for diarrheal disease. In Kenya, only the water treatment arm reduced *E. coli* levels in stored drinking water, and no WASH intervention reduced *E. coli* levels on children’s hands or on sentinel objects [27]. In Bangladesh, two studies were conducted: the first, four months after the intervention, sampled drinking and ambient water, children’s hands, food given to young children, courtyard soil and flies, in the sanitation only and combined WASH arms [26]; the second, 12 and 24 months after the intervention, sampled drinking water at source and as stored, children’s hands, children’s food and sentinel objects [25]. In the first of these two studies [26], the prevalence of *E. coli* in stored water was reduced only in the combined WASH arm (prevalence ratio [PR] 0.38; 95% CI:0.32–0.44), with no effect on any other sampled pathway (soil, hygiene, flies or food). In the second study, the prevalence of *E. coli* in stored drinking water was reduced by the water treatment only intervention (PR 0.62; 95% CI 0.53–0.72) and combined WASH intervention (PR 0.75; 95% CI 0.69–0.81), and the prevalence of *E. coli* in food was reduced in the single water treatment arm (PR 0.70; 95% CI 0.57–0.86), the single handwashing (PR 0.68; 95% CI 0.56–0.83) and combined WASH interventions (PR 0.89; 95% CI: 0.78–1.01) [25].

Against a low baseline prevalence of diarrhea, and the limited environmental impact of the WASH interventions evaluated, it is notable that a 40% relative reduction in diarrheal disease prevalence was achieved in Bangladesh (an absolute reduction of approximately two percentage points compared to a one-week prevalence of 5.9% in the control arm) [1]. This result was strengthened by a separate comparison of the prevalance of giardiasis across study arms which also showed a marked reduction in infections among all WASH arms except water chlorination [28]. In both the Kenya and Bangladesh trials, which included chlorination only intervention arms, bacterial contamination of stored drinking water was reduced in this arm, but there was no effect on diarrheal disease. As discussed above, this may be due to the resistance of certain diarrhegenic pathogens’ resistance to chlorine, e.g. *Cryptosporidium* and *Giardia* [29].

These results suggest that, in settings such as these, more comprehensive or ambitious interventions may be needed to achieve a major impact on child health. In different settings, with more limited WASH conditions – for example, where most people practice open defecation or rely on untreated surface water – these interventions may still yield benefits. Alternatively, in similar settings, more ambitious interventions that address other potentially important exposure sources and/or routes, such as animal waste or foodborne transmission, may be effective in reducing diarrheal disease.

### 3. Historically, large, population-level gains in child health have not been achieved without significant improvements in WASH services

Globally, as countries and regions have transitioned from scenarios in which most of the population have limited or basic WASH services, to one in which most have access to safely managed services, there have been large coincident improvements in public health. Often these improvements have been dramatic with regards to child health and mortality, specifically [30–32]. They have commonly been associated with major improvements in water and sanitation infrastructure akin to the SDG category of ‘safely managed services’ - that is ensuring a piped supply of safe drinking water directly to the household, or reticulated transportation of human waste to treatment facilities – rather than the more modest changes in service access evaluated in these trials.

Typically, these changes took place over decades. For example, in Victorian Britain, while the great municipal water reforms began in the 1840s, it was not until the 1870s that major investments in sewered household connections began in most cities [32]. Changes in diarrheal disease mortality followed slowly. In London, for example, infantile diarrheal disease mortality was still rising in 1900. In fact, trends in child and infant mortality suggest that benefits accrue from incremental and progressive steps over time, and that major health dividends may come late in that process, as was the case in England and Wales (1900–1920) [32] or in the USA (1920–1930) [31]. Specific innovations are sometimes credited with these health benefits – e.g., in the USA, disinfecting water supplies coincided with major health gains – but crediting these health improvements to water treatment alone ignores, for example, the fact that the infrastructure for the distribution of this treated water was already in place. The lesson perhaps lies in not seeking to attribute benefits to individual WASH factors but in that the public health dividends are paid when comprehensive services are in place, as now envisaged under the new SDG.

These new studies do not challenge the general view that large-scale improvements in water and sanitation infrastructure played an important historical role in improving child health in high-income countries (HIC). The interventions coinciding with dramatic improvements in child health in many of these HICs represented decades of large-scale public investment in piped drinking water and sewered sanitation, as opposed to the provision of basic pit latrines, point-of-use chlorination of water, and handwashing stations as evaluated under these trials.

### 4. Current evidence suggests that basic WASH services alone are unlikely to have a large impact on childhood stunting

A Cochrane Review published in 2013, which addressed the effect of WASH interventions on linear growth, identified very few rigorous studies. It concluded there was, “weak evidence of a borderline statistically significant small effect of 0.08 HAZ” [33]. Subsequent intervention studies have produced mixed results, from significant improvements in linear growth [34, 35] to no effect [36–38]. The new trials considered here hypothesised that WASH interventions might improve linear growth among children by reducing symptomatic and asymptomatic enteric infections, with this effect mediated at least in part by environmental enteric dysfunction (EED) [5, 6], a subclinical condition affecting gut structure and function [39]. The reported effects on symptomatic enteric infections – that is, caregiver-reported diarrheal disease – were mixed, as discussed above, but all three studies found no effect of any WASH intervention arm on linear growth. Furthermore, these factorial studies were specifically designed to assess the combined and independent effects of WASH and nutrition interventions on linear growth, and found no additive benefit of these basic WASH interventions versus nutrition (that is, improved infant and young child feeding, including daily, small-quantity lipid-based nutrient supplements during the period of complementary feeding), alone.

If symptomatic and asymptomatic enteric infection contributes to linear growth faltering, as hypothesized by these studies [5, 6], these results suggest that preventing these infections is unlikely to be achieved with low-cost basic household interventions in highly contaminated settings where young children are exposed to enteric pathogens repeatedly and via multiple routes. To what extent more intensive WASH interventions – such as providing a microbially safe and continuous supply of drinking water piped to the household, or a community-level sewered sanitation system – might impact childhood stunting remains an open question that has so far not been addressed by rigorous trials [33]. The lack of experimental studies of such interventions reflects multiple inherent challenges, including the difficulty of randomly allocating networked infrastructure; the generally high levels of population movement in urban areas; and the long follow-up and large sample sizes required to study linear growth among children. These challenges are not insurmountable, but are certainly formidable.

The results reinforce a well-established view in the nutrition sector that tackling a multifactorial chronic condition such as stunting requires broad and sustained action at multiple levels and across different sectors [40]. The postnatal nutrient supplementation and complementary feeding behaviour change interventions in these same trials, achieved only modest gains in linear growth, despite very high and sustained compliance. These effects are consistent with the wider literature [41, 42], further demonstrating that the underlying causes of stunting remain remarkably poorly understood, and that population-level reductions likely require broad strategies to increase both the availability of nutrients and to reduce nutrient malabsorption. The experience of Brazil, where a dramatic decline in stunting has been achieved, is of three decades of sustained action across multiple sectors, including food, health, social protection as well as water and sanitation [43].

### 5. The results of these trials do not undermine the new and ambitious SDG target of safely managed services for all

In summary, the basic, household-level interventions evaluated under these trials did not test the ambitious new SDG targets of universal access to safely managed water and sanitation, and therefore do not provide evidence for or against this level of service. The interventions evaluated in these trials effected only modest changes with regard to the ‘WASH ladder’ concept that has been developed for tracking progress against the SDG targets [7]. This ladder represents incremental gains in the quality of WASH services, and there is some evidence to support the assumption that, as the quality of services improve, so too does the magnitude of effect on diarrheal disease. However, the evidence for higher levels of service (e.g., piped drinking water and sewered sanitation connections) is generally of low quality [44].

The ‘sanitation’ interventions included in these trials did not seek to “end open defecation” at a community level, which is a key component of the WASH SDG (SDG 6.2). Instead, the trials focused on improving sanitation at the level of the child’s household, or immediately around the household, as earlier formative work suggested that children’s environmental exposure to enteropathogens occurred within the household or the immediate compound domains [45, 46]. The effect of these interventions on community-level sanitation was, therefore, limited. In fact, at baseline, in both Bangladesh and Kenya, access to household sanitation facilities was relatively high pre-intervention.

The ‘water’ interventions in these trials sought only to improve the microbial quality of drinking water drawn from existing water sources by promoting chlorination in the household. Under the WASH SDG, safely managed drinking water is defined as, “drinking water from an improved water source that is located on premises, available when needed and free from fecal and priority chemical contamination” (SDG 6.1) [7]. In these trials, the distribution of drinking water was not changed to bring water sources closer to the household, to limit service disruptions to ensure water was available when needed, and chemical or microbial contamination of drinking water at source was not addressed. In relation to how water influences disease transmission, an old distinction can be drawn between ‘waterborne’ transmission, that is where transmission occurs via ingestion of water containing pathogens, and ‘water-washed’ transmission, wherein person-to-person transmission results from insufficient water to practice adequate personal and domestic hygiene [47, 48]. It has been observed that when the household water supply is on-plot or piped directly into the household, the amount of water consumed increases dramatically [49], and to a level wherein adequate water is available to meet hygiene needs and reduce health risks [50, 51]. Critically, then, neither the distance to water source nor the volume of water consumed was changed by these interventions.

These trials did not evaluate the effect of safely managed water and community-level safely managed sanitation services, as called for under the new WASH SDG, on child stunting or diarrhea. The basic interventions evaluated in these trials were seemingly insufficient to comprehensively reduce enteric pathogen exposure, and had mixed effects on diarrhea. This may, in part, explain the lack of effect on stunting, if diarrhea mediates at least some part of this relationship.

## Conclusions

These three trials evaluated similar WASH interventions in low-income rural settings and found no effect on childhood stunting and mixed effects on childhood diarrheal disease. Conventionally, WASH interventions are understood to act on these health outcomes by changing infrastructure and/or behaviors to limit environmental exposure to infectious agents. The interventions assessed under these three major trials were relatively successful in the first stage of changing infrastructure and/or behaviours, but seemingly failed to sufficiently reduce environmental exposure to enteric pathogens to improve linear childhood growth.

While randomization offers clear advantages with regards to internal validity, this can come at the cost of external validity and generalizability [52]. In clinical studies, the observed relationship between intervention and outcome can often be reasonably assumed to hold constant over time, population and setting. The same should not be assumed for complex public health interventions that interact powerfully with external contextual factors, which may diminish or potentiate effects. However, that these three studies evaluated the same interventions under similar protocols in three different settings sheds at least some light on the generalizability of findings and potential sources of observed heterogeneity.

These results warrant attention because basic WASH interventions similar to these are often deployed in low-income rural settings with the expectation of improving child health, although this is rarely the sole justification. At the same time, these interventions did not address common features of national WASH policies; i.e., drinking water supply or distribution, and community-level sanitation. As an example of this, the current national WASH policies of all three countries (Bangladesh, Kenya and Zimbabwe) where these studies took place aim to end open defecation, and to expand, repair or rehabilitate rural water supplies. At a global level, too, these aspects of WASH not addressed in these trials are central to the new SDG, with its objectives of ending open defecation and ensuring universal access to safely managed drinking water [7].

WASH trials often produce heterogenous results, reflecting the inherent complexity of interventions combining infrastructure and behavior, and which interact strongly with specific, local environmental and social systems. Indeed, the mixed results for diarrheal disease reported across these three trials of very similar interventions bear witness to this. Our view is that these three new trials do not show that WASH in general cannot influence child linear growth, but rather that these specific interventions failed to do so in settings where stunting remains an important public health challenge. These findings warrant the attention of policy-makers and practitioners, and should give some pause for reflection with regards to the design of programs in low-income rural settings that include such low-cost WASH interventions with the goal of improving child growth and reducing diarrhea.

With growing evidence of the burden of enteric pathogen carriage, and the associated growth and developmental consequences in low-income settings [11, 12], calls have been made for ‘transformative WASH’ [53] or ‘WASH++’ interventions [54]. Although not clearly defined, nor as yet evaluated, we support this call for transformative WASH because it encapsulates the guiding principle that – in any context – what is needed is a comprehensive package of interventions tailored to address the local exposure landscape and enteric disease burden.

## Additional file

Additional file 1: Table providing full details of Cochrane risk of bias assessment for the WASH-Benefits and SHINE trials (Word document). (DOCX 18 kb)

## Data Availability

Not applicable.

## Abbreviations

EED: Environmental enteric dysfunction
WASH: Water, sanitation and hygiene
LIC: Low-income countries
LMIC: Low and middle-income countries
HIC: High-income countries
WHO: World Health Organization
BMGF: Bill and Melinda Gates Foundation
SDG: Sustainable Development Goal

## References

[1] Luby SP, Rahman M, Arnold BF, Unicomb L, Ashraf S, Winch PJ (2018). Effects of water quality, sanitation, handwashing, and nutritional interventions on diarrhoea and child growth in rural Bangladesh: a cluster randomised controlled trial. Lancet Glob Health.

[2] Null C, Stewart CP, Pickering AJ, Dentz HN, Arnold BF, Arnold CD (2018). Effects of water quality, sanitation, handwashing, and nutritional interventions on diarrhoea and child growth in rural Kenya: a cluster-randomised controlled trial. Lancet Glob Health.

[3] Humphrey JH, Mbuya MN, Ntozini R, Moulton LH, Stoltzfus RJ, Tavengwa NV (2019). Independent and combined effects of improved water, sanitation, and hygiene, and improved complementary feeding, on child stunting and anaemia in rural Zimbabwe: a cluster-randomised trial. Lancet Glob Health.

[4] Schulz KF, Altman DG, Moher D (2010). CONSORT 2010 statement: updated guidelines for reporting parallel group randomised trials. BMC Med.

[5] Arnold BF, Null C, Luby SP, Unicomb L, Stewart CP, Dewey KG (2013). Cluster-randomised controlled trials of individual and combined water, sanitation, hygiene and nutritional interventions in rural Bangladesh and Kenya: the WASH benefits study design and rationale. BMJ Open.

[6] Humphrey JH, Jones AD, Manges A, Mangwadu G, Maluccio JA, Mbuya MN (2015). The sanitation hygiene infant nutrition efficacy (SHINE) trial: rationale, design, and methods. Clin Infect Dis.

[7] World Health Organization (WHO), United Nations Children’s Fund (UNICEF) (2017). Progress on drinking water, sanitation and hygiene: 2017 update and SDG baselines.

[8] World Health Organization (WHO), United Nations Children’s Fund (UNICEF) (2015). Improving nutritional outcomes with better water, sanitation and hygiene: practical solutions for policies and programmes.

[9] Freeman MC, Ogden S, Jacobson J, Abbott D, Addiss DG, Amnie AG (2013). Integration of water, sanitation, and hygiene for the prevention and control of neglected tropical diseases: a rationale for inter-sectoral collaboration. PLoS Negl Trop Dis.

[10] Velleman Y, Mason E, Graham W, Benova L, Chopra M, Campbell OM (2014). From joint thinking to joint action: a call to action on improving water, sanitation, and hygiene for maternal and newborn health. PLoS Med.

[11] Liu J, Platts-Mills JA, Juma J, Kabir F, Nkeze J, Okoi C (2016). Use of quantitative molecular diagnostic methods to identify causes of diarrhoea in children: a reanalysis of the GEMS case-control study. Lancet..

[12] Platts-Mills JA, Liu J, Rogawski ET, Kabir F, Lertsethtakarn P, Siguas M (2018). Use of quantitative molecular diagnostic methods to assess the aetiology, burden, and clinical characteristics of diarrhoea in children in low-resource settings: a reanalysis of the MAL-ED cohort study. Lancet Glob Health.

[13] Mac Kenzie WR, Hoxie NJ, Proctor ME, Gradus MS, Blair KA, Peterson DE (1994). A massive outbreak in Milwaukee of Cryptosporidium infection transmitted through the public water supply. N Engl J Med.

[14] Dillingham RA, Lima AA, Guerrant RL (2002). Cryptosporidiosis: epidemiology and impact. Microbes Infect.

[15] Bradley DJ, Emurwon P (1968). Predicting the epidemiological effects of changing water sources. I. a quantitative approach. East Afr Med J.

[16] Kawata K (1978). Water and other environmental interventions--the minimum investment concept. Am J Clin Nutr.

[17] Briscoe J (1984). Intervention studies and the defintiion of dominant transmission routes. Am J Epidemiol.

[18] Shuval HI, Tilden RL, Perry BH, Grosse RN (1981). Effect of investments in water supply and sanitation on health status: a threshold-saturation theory. Bull World Health Organ.

[19] Ashbolt NJ (2004). Microbial contamination of drinking water and disease outcomes in developing regions. Toxicology..

[20] Cash RA, Music SI, Libonati JP, Craig JP, Pierce NF, Hornick RB (1974). Response of man to infection with Vibrio cholerae. II. Protection from illness afforded by previous disease and vaccine. J Infect Dis.

[21] DuPont HL, Hornick RB, Dawkins AT, Snyder MJ, Formal SB (1969). The response of man to virulent Shigella flexneri 2a. J Infect Dis.

[22] Black RE, Levine MM, Clements ML, Hughes TP, Blaser MJ (1988). Experimental campylobacter jejuni infection in humans. J Infect Dis.

[23] Wagner E, Lanoix J (1958). Excreta disposal for rural areas and small communities. Monogr Ser World Health Organ.

[24] Wolf J, Johnston R, Hunter P, Gordon B, Medlicott K, Prüss-Ustün A (2019). A Faecal contamination index for interpreting heterogeneous diarrhoea impacts of water, sanitation and hygiene interventions and overall, regional and country estimates of community sanitation coverage with a focus on low- and middle-income countries. Int J Hyg Environ Health.

[25] Ercumen A, Mertens A, Arnold BF, Benjamin-Chung J, Hubbard AE, Ahmed MA (2018). Effects of single and combined water, sanitation and handwashing interventions on fecal contamination in the domestic environment: a cluster-randomized controlled trial in rural Bangladesh. Environ Sci Technol.

[26] Ercumen A, Pickering AJ, Kwong LH, Mertens A, Arnold BF, Benjamin-Chung J (2018). Do sanitation improvements reduce fecal contamination of water, hands, food, soil, and flies? Evidence from a cluster-randomized controlled trial in rural Bangladesh. Environ Sci Technol.

[27] Pickering A, Swarthout J, Mureithi M, Mboya J, Arnold B, Wolfe M (2019). Can individual and integrated water, sanitation, and handwashing interventions reduce fecal contamination in the household environment? Evidence from the WASH Benefits cluster-randomized trial in rural Kenya.

[28] Lin A, Ercumen A, Benjamin-Chung J, Arnold BF, Das S, Haque R (2018). Effects of water, sanitation, handwashing, and nutritional interventions on child enteric protozoan infections in rural Bangladesh: a cluster-randomized controlled trial. Clin Infect Dis.

[29] Betancourt WQ, Rose JB (2004). Drinking water treatment processes for removal of Cryptosporidium and Giardia. Vet Parasitol.

[30] Alsan M, Goldin C (2018). Watersheds in child mortality: the role of effective water and sewerage infrastructure, 1880 to 1920.

[31] Cutler D, Deaton A, Lleras-Muney A (2006). The determinants of mortality. J Econ Perspect.

[32] Bell F, Millward R (1998). Public health expenditures and mortality in England and Wales, 1870–1914. Contin Chang.

[33] Dangour Alan D, Watson L, Cumming O, Boisson S, Che Y, Velleman Y (2013). Interventions to improve water quality and supply, sanitation and hygiene practices, and their effects on the nutritional status of children. Cochrane Database Syst Rev.

[34] Pickering AJ, Djebbari H, Lopez C, Coulibaly M, Alzua ML (2015). Effect of a community-led sanitation intervention on child diarrhoea and child growth in rural Mali: a cluster-randomised controlled trial. Lancet Glob Health.

[35] Hammer J, Spears D (2016). Village sanitation and child health: effects and external validity in a randomized field experiment in rural India. J Health Econ.

[36] Patil SR, Arnold BF, Salvatore AL, Briceno B, Ganguly S, Colford JM (2014). The effect of India's total sanitation campaign on defecation behaviors and child health in rural Madhya Pradesh: a cluster randomized controlled trial. PLoS Med.

[37] Clasen T, Boisson S, Routray P, Torondel B, Bell M, Cumming O (2014). Effectiveness of a rural sanitation programme on diarrhoea, soil-transmitted helminth infection, and child malnutrition in Odisha, India: a cluster-randomised trial. Lancet Glob Health.

[38] Sinharoy SS, Schmidt W-P, Wendt R, Mfura L, Crossett E, Grépin KA (2017). Effect of community health clubs on child diarrhoea in western Rwanda: cluster-randomised controlled trial. Lancet Glob Health.

[39] Keusch GT, Denno DM, Black RE, Duggan C, Guerrant RL, Lavery JV (2014). Environmental enteric dysfunction: pathogenesis, diagnosis, and clinical consequences. Clin Infect Dis.

[40] Bhutta ZA, Das JK, Rizvi A, Gaffey MF, Walker N, Horton S (2013). Evidence-based interventions for improvement of maternal and child nutrition: what can be done and at what cost?. Lancet..

[41] Panjwani A, Heidkamp R (2017). Complementary feeding interventions have a small but significant impact on linear and ponderal growth of children in low- and middle-income countries: a systematic review and meta-analysis. J Nutr.

[42] Dewey KG, Adu-Afarwuah S (2008). Systematic review of the efficacy and effectiveness of complementary feeding interventions in developing countries. Matern Child Nutr.

[43] KonnoI SC, de LimaII ALL, CondeI WL (2009). Causes for the decline in child under-nutrition in Brazil, 1996–2007. Rev Saúde Pública.

[44] Wolf J, Hunter PR, Freeman MC, Cumming O, Clasen T, Bartram J (2018). Impact of drinking water, sanitation and hand washing with soap on childhood diarrhoeal disease: updated meta-analysis and meta-regression. Tropical Med Int Health.

[45] Ngure FM, Humphrey JH, Mbuya MN, Majo F, Mutasa K, Govha M (2013). Formative research on hygiene behaviors and geophagy among infants and young children and implications of exposure to fecal bacteria. Am J Trop Med Hyg.

[46] Kwong LH, Ercumen A, Pickering AJ, Unicomb L, Davis J, Luby SP (2016). Hand-and object-mouthing of rural Bangladeshi children 3–18 months old. Int J Environ Res Public Health.

[47] Bradley D, Feachem RG, McGarry MG, Mara DD (1977). Health aspects of water supplies in tropical countries. Water, wastes and health in hot climates.

[48] White GF, Bradley DJ, White AU, Ahmed T (1972). Drawers of water.

[49] Cairncross S, Feachem RG (1983). Environmental health engineering in the tropics: an introductory text.

[50] Howard G, Bartram J (2003). Domestic water quantity, service level and health.

[51] Pickering AJ, Davis J (2012). Freshwater availability and water fetching distance affect child health in sub-Saharan Africa. Environ Sci Technol.

[52] Deaton A, Cartwright N (2018). Understanding and misunderstanding randomized controlled trials. Soc Sci Med.

[53] Pickering AJ, Null C, Winch PJ, Mangwadu G, Arnold BF, Prendergast AJ (2019). The WASH benefits and SHINE trials: interpretation of WASH intervention effects on linear growth and diarrhoea. Lancet Glob Health.

[54] Husseini M, Darboe MK, Moore SE, Nabwera HM, Prentice AM (2018). Thresholds of socio-economic and environmental conditions necessary to escape from childhood malnutrition: a natural experiment in rural Gambia. BMC Med.

