# Supplementary material for: The implications of three major new trials for the effect of water, sanitation and hygiene on childhood diarrhea and stunting: a consensus statement
Source: BMC Med. 2019 Aug 28;17:173. doi: 10.1186/s12916-019-1410-x (PMC6712663; doi:10.1186/s12916-019-1410-x)
Supplement: Supplementary file 1 — Table providing full details of Cochrane risk of bias assessment for the WASH-Benefits and SHINE trials (Word document). (DOCX 18 kb) [file 12916_2019_1410_MOESM1_ESM.docx]

Additional File 1. Table providing full details of Cochrane Risk of Bias assessment for the WASH-B and SHINE trials

| **Study** | | **Random sequence generation (selection bias)** | **Allocation concealment (selection bias)** | **Selective Reporting (reporting bias)** | **Blinding of participants and personnel (performance bias)** | **Blinding of outcome assessment (detection bias)** | **Incomplete outcome data (attrition bias)** | **Other bias** |
| --- | --- | --- | --- | --- | --- | --- | --- | --- |
| **Humphrey et al.,**  **2019^3^** | **Risk level** | Low | Low | Low | High | High | Low risk | Low |
|  | **Reason** | Constrained randomisation | Central randomization | Study protocol available and prespecified outcomes reported | Participants and fieldworkers not blinded | Blinding not possible for data collectors | Loss to follow up reported, intention-to-treat analysis |  |
| **Luby et al.,**  **2018^1^** | **Risk level** | Low | Low | Low | High | High | Low | Low |
|  | **Reason** | Block randomization with random number generator | Central randomization | Study protocol available and prespecified outcomes reported | Participants not blinded | Blinding not possible for data collectors | Loss to follow up reported, intention-to-treat analysis |  |
| **Null et al.,**  **2018^2^** | **Risk level** | Low | Low | Low | High | High | Low | Low |
|  | **Reason** | Block randomization with random number generator | Central randomization | Study protocol available and prespecified outcomes reported | Participants not blinded | Not possible to blind data collectors’ inferred treatment status if they saw interventions in study communities | Loss to follow up reported, intention-to-treat analysis |  |
